# Global trends in minipuberty research: A comprehensive bibliometric analysis

**DOI:** 10.1097/MD.0000000000048309

**Published:** 2026-05-15

**Authors:** Yi Jiang, Kaiqin Jin, Wen Zhong, Xiaozhu Shen, Xiaojing Zhong

**Affiliations:** a Department of General Medicine, Huzhou Central Hospital, Fifth School of Clinical Medicine of Zhejiang Chinese Medical University, Huzhou, China; b The Second Department of Critical Care Medicine, The Second Affiliated Hospital of Anhui Medical University, Hefei, China; c Department of Geriatrics, Lianyungang Hospital Affiliated to Jiangsu University (Lianyungang Second People’s Hospital), Lianyungang, China; d Department of Endocrinology, Huzhou Central Hospital, Fifth School of Clinical Medicine of Zhejiang Chinese Medical University, Huzhou, China.

**Keywords:** bibliometric analysis, developmental trends, endocrine research, HPG axis, minipuberty

## Abstract

**Background:**

Minipuberty represents a critical window of transient postnatal hypothalamic-pituitary-gonadal axis activation, yet its global research landscape has not been systematically mapped. This study aims to perform a comprehensive bibliometric analysis to delineate the evolving research landscape, collaborative networks, and thematic shifts in minipuberty research over the past two decades.

**Methods:**

Publications from 2003 to 2025 were retrieved from the Web of Science Core Collection. A final corpus of 201 articles underwent analysis using Bibliometrix, VOSviewer, and CiteSpace to examine publication trends, country/institution contributions, co-authorship networks, and keyword dynamics.

**Results:**

Annual publication output increased markedly after 2013, peaking in 2024. Denmark and the United States were the most productive countries, with the University of Copenhagen as the leading institution. Thematic focus evolved from foundational studies on adrenal spermatogonia and fertility to contemporary investigations into endocrine disruption, therapeutic interventions, and genetic regulation. The *Journal of Clinical Endocrinology & Metabolism* was the most prolific journal. Seminal, highly cited works established normative hormonal profiles and clinical links to disorders such as cryptorchidism.

**Conclusion:**

Minipuberty research has progressed from descriptive studies to translational science, with emerging trends emphasizing molecular mechanisms and long-term developmental outcomes. Future work should leverage longitudinal cohorts, advanced biomolecular techniques, and interdisciplinary collaboration to fully elucidate this critical developmental window.

## 1. Introduction

Minipuberty describes the transient activation of the hypothalamic–pituitary–gonadal (HPG) axis occurring shortly after birth, characterized by a surge in gonadotropin and sex steroid levels.^[1]^ This developmental window is critical for early sexual differentiation, genital development, and the establishment of future reproductive function.^[2–5]^ It influences not only reproductive organ maturation but also infant body composition and aspects of neurodevelopment.^[6,7]^

Despite its physiological importance, minipuberty remains a relatively understudied area within endocrinology and developmental biology. Research has expanded gradually since the early 2000s,^[8]^ yet a comprehensive synthesis of the field’s evolution, key contributors, and emerging trends is lacking. Recent advances in bibliometric analysis provide powerful tools for mapping the development of scientific fields, identifying research trends, and highlighting knowledge gaps.

This study aims to conduct a comprehensive bibliometric analysis of global minipuberty research. We seek to trace its temporal development, identify influential studies and authors, illustrate geographical and institutional collaboration patterns, and outline thematic shifts. Through this approach, we aim to provide a clear landscape of the current state of knowledge, reveal research gaps, and propose future directions to foster increased interest and collaboration in this significant field.

## 2. Methods

### 2.1. Data source

This study utilized bibliometric data from the **Web of Science Core Collection**, the standard database for such analyses, as it provides the standardized citation data and complete metadata required for network analysis. To ensure methodological consistency and avoid biases from differing database indexing practices, we focused exclusively on WoSCC – a common approach in bibliometrics sufficient for mapping the core research landscape and collaboration patterns in this field.

### 2.2. Search strategy

A systematic search was conducted on June 1, 2025, using the following Boolean query in the WoSCC Advanced Search interface: TS=((“mini puberty”) OR (minipuberty) OR (“mini-puberty”)) AND DT=(Article OR Review).

### 2.3. Literature screening

#### 2.3.1. Screening for relevance

The remaining unique records underwent a 3-step, independent screening by 2 researchers (YJ and KQJ): title screening, abstract screening, and full-text assessment.

#### 2.3.2. Verification and consensus

At each stage, **any discrepancy between the 2 reviewers was resolved through discussion until consensus was reached.** This dual-reviewer process with consensus resolution served as our verification mechanism, ensuring the reliability and reproducibility of the inclusion/exclusion decisions.

#### 2.3.3. Process documentation

**Figure 1.**
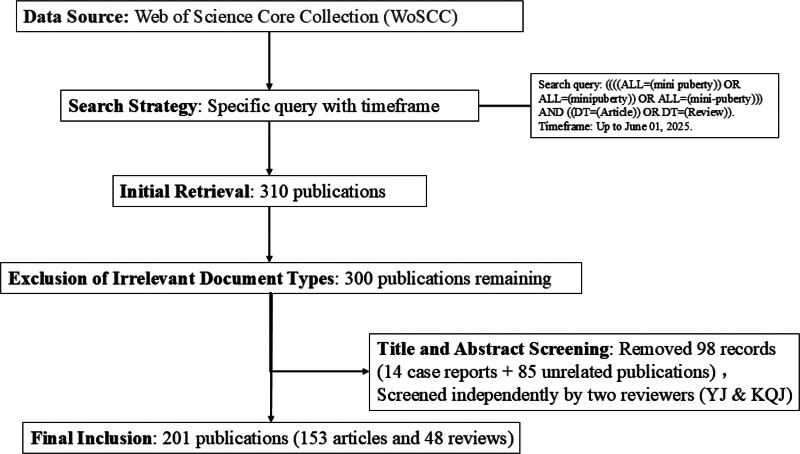
Flowchart of literature search and screening.

The complete flow of this process, including the number of records at each stage, is clearly illustrated in Figure 1.

### 2.4. Bibliometric analysis

Data analysis was performed using **R (version 4.5.0; Vienna, Austria)** with the **bibliometrix package**,^[9]^ **CiteSpace (version 6.4.R1; Drexel University, Philadelphia**), and **VOSviewer (version 1.6.20; Leiden University, Netherlands)**. Collaboration network maps (country, institution, author) were generated using VOSviewer. For co-authorship analysis, the full counting method was used. The minimum number of documents was set to 1 for an item to be included in the network. Keyword co-occurrence, clustering, and burst detection analyses were performed in CiteSpace. Parameters were configured as follows: time slicing from 2003 to 2025 (1 year per slice); term sources selected from Title, Abstract, Author Keywords, and Keywords Plus; node type as Keyword; selection criteria using the g-index (*k* = 25) for term extraction; and pruning of the merged network using the Pathfinder algorithm. The analysis emphasized transparency and reproducibility.

## 3. Results

### 3.1. Data retrieval and screening process

The literature search was restricted to articles and reviews published until June 1, 2025, yielding an initial total of 310 records. To ensure relevance and quality, retracted publications, conference proceedings, and early-access articles were excluded, resulting in 300 records eligible for screening. Two independent investigators (Jiang and Jin) evaluated the titles, abstracts, and full texts of these records. Through this process, 14 case reports and 85 articles not pertinent to minipuberty were removed.

Following rigorous screening, a final corpus of 201 publications was included for bibliometric analysis, comprising 154 research articles and 47 reviews. The screening procedure is detailed in Figure 1.

**Figure 2.**
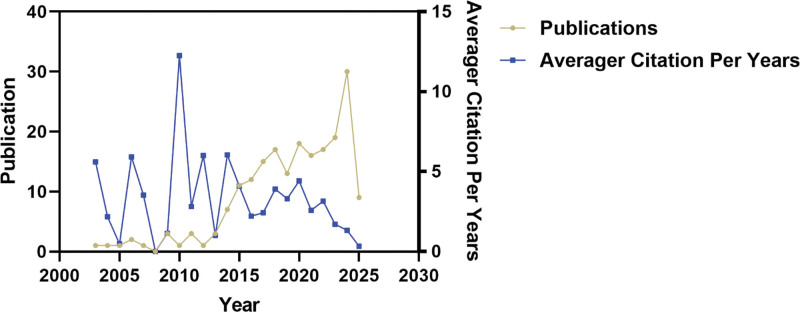
Annual number of publications on minipuberty (2003–2025).

Between 2003 and 2025, research in this field involved 867 authors from 27 countries and regions, affiliated with 331 organizations. These studies were published across 91 journals, with an international collaboration rate of 33.66% and an average of 22.53 citations per article. As depicted in Figure 2, interest in minipuberty research grew noticeably starting in 2013, with a rapid increase in annual publications – reflecting an average growth rate of 10.5% from 2013 to 2024. The year 2024 marked the highest output, with 30 articles published. This growth phase, beginning around 2013, may correspond with broader clinical recognition of the significance of minipuberty and advancements in sensitive hormonal assay technologies.

### 3.2. Country analysis

**Figure 3.**
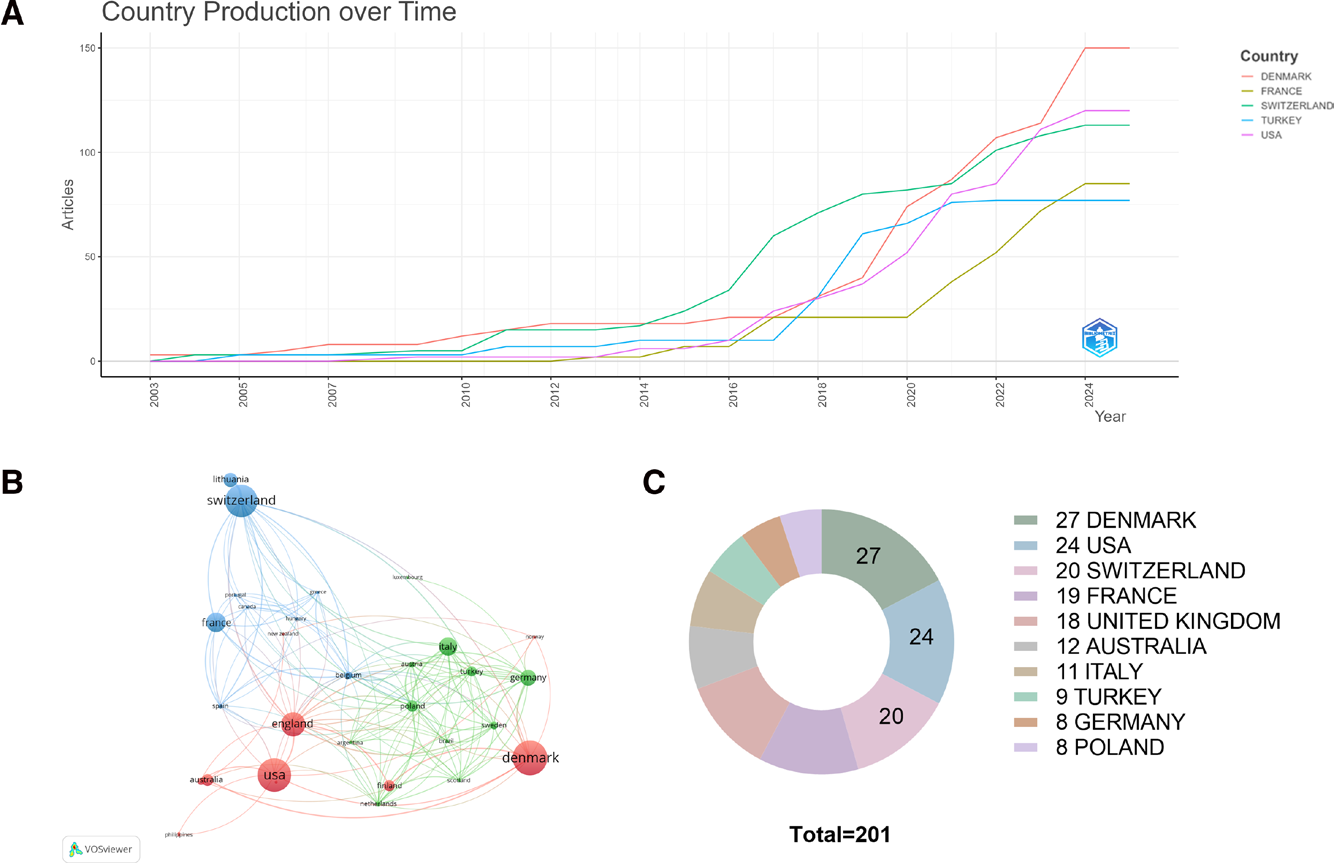
Country-level contributions. (A) Publication trends of the top 5 countries over time. (B) Global collaboration network map. (C) Publication share of the top 10 countries.

Figure 3A displays the publication output over time for the top 5 countries, indicating a notable surge starting in 2016, during which Switzerland led in productivity. By 2024, Denmark had become the leading contributor. Figure 3B presents the global distribution of publications and international collaborative networks. Corresponding author affiliations indicate that Denmark and the United States produced the highest number of publications, with Denmark contributing 27 articles, followed by the United States with 24. As summarized in Table 1 and Figure 3C, the top 10 countries/regions are ranked according to their share of total publications. The average citation per publication (ACPP) metric reflects the academic impact of publications from each country. Denmark ranks fifth in ACPP, while the United States and Switzerland rank 14th and 10th, respectively. These findings highlight Denmark and the United Kingdom as major contributors in terms of both output and influence. Notably, although the United Kingdom ranks fifth in total publications, it holds the highest ACPP among the top contributors. This suggests that British research in this field, though less voluminous, has produced publications with substantial academic impact, potentially due to influential reviews or pivotal longitudinal cohort studies.

**Table 1:** Top 10 countries/regions by number of publications.

Country	Publications	SCP	MCP	MCP %	Citations	ACPP	Rank of ACPP
Denmark	27	16	11	40.7	1101	40.80	5
United States	24	19	5	20.8	311	13.00	13
Switzerland	20	10	10	50	393	19.60	10
France	19	15	4	21.1	268	14.10	12
The United Kingdom	18	10	8	44.4	945	52.50	4
Australia	11	4	7	63.6	112	10.20	17
Italy	11	11	0	0	308	28.00	7
Turkey	9	9	0	0	72	8.00	23
Germany	8	5	3	37.5	184	23.00	8
Poland	8	4	4	50	27	3.40	24

ACPP = average citation per publication, MCP = multiple country publication, SCP = single country publication.

### 3.3. Organization analysis

**Figure 4.**
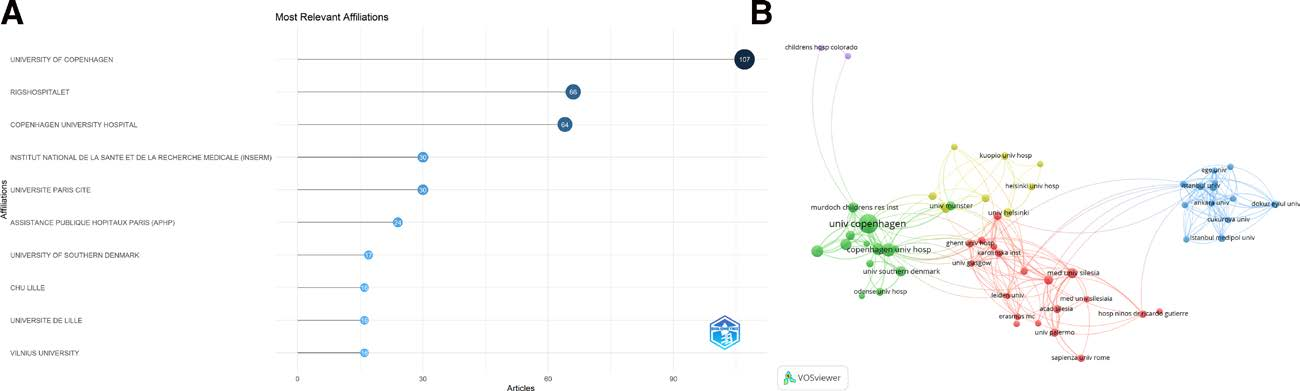
Institutional contributions and collaboration. (A) Top 10 institutions by publication count. (B) Institutional collaboration network.

A total of 341 organizations contributed to research in this field. As shown in Figure 4A, the 10 most productive institutions were: the University of Copenhagen, Rigshospitalet, Copenhagen University Hospital, Institut National de la Santé et de la Recherche Médicale, Université Paris Cité, Assistance Publique–Hôpitaux de Paris, the University of Southern Denmark, CHU Lille, Université de Lille, and Vilnius University. Among these, 5 are based in Denmark, 4 in France, and 1 in Lithuania. Collaborative networks among the most prolific institutions are illustrated in Figure 4B.

### 3.4. Journal analysis

A total of 201 articles on minipuberty, which are indexed in the Science Citation Index , were published across 91 journals. Among these, 24 journals contributed more than 3 publications each. As summarized in Table 2, the 10 most prolific journals are listed, with the Journal of Clinical Endocrinology & Metabolism being the leading outlet, publishing 16 articles. The overall quality of journals in this field is notable, with 5 ranked within the Q1 or Q2 quartiles of the Journal Citation Reports, and the highest journal impact factor reaching 6.

**Table 2:** Top 10 most productive journals.

Sources	Articles	JCR	Impact factor
Journal of Clinical Endocrinology & Metabolism	16	1	5.1
Frontiers in Endocrinology	10	1	4.6
Clinical Endocrinology	8	3	3
Hormone Research in Paediatrics	7	3	2.7
Sexual Development	7	2	2.4
Annales d’Endocrinologie	6	3	2.9
Journal of Pediatric Urology	6	2	2
Basic and Clinical Andrology	5	3	2
Journal of Pediatric Endocrinology & Metabolism	5	4	1.3
Human Reproduction	4	1	6

JCR = Journal Citation Reports.

### 3.5. Author analysis

**Figure 5.**
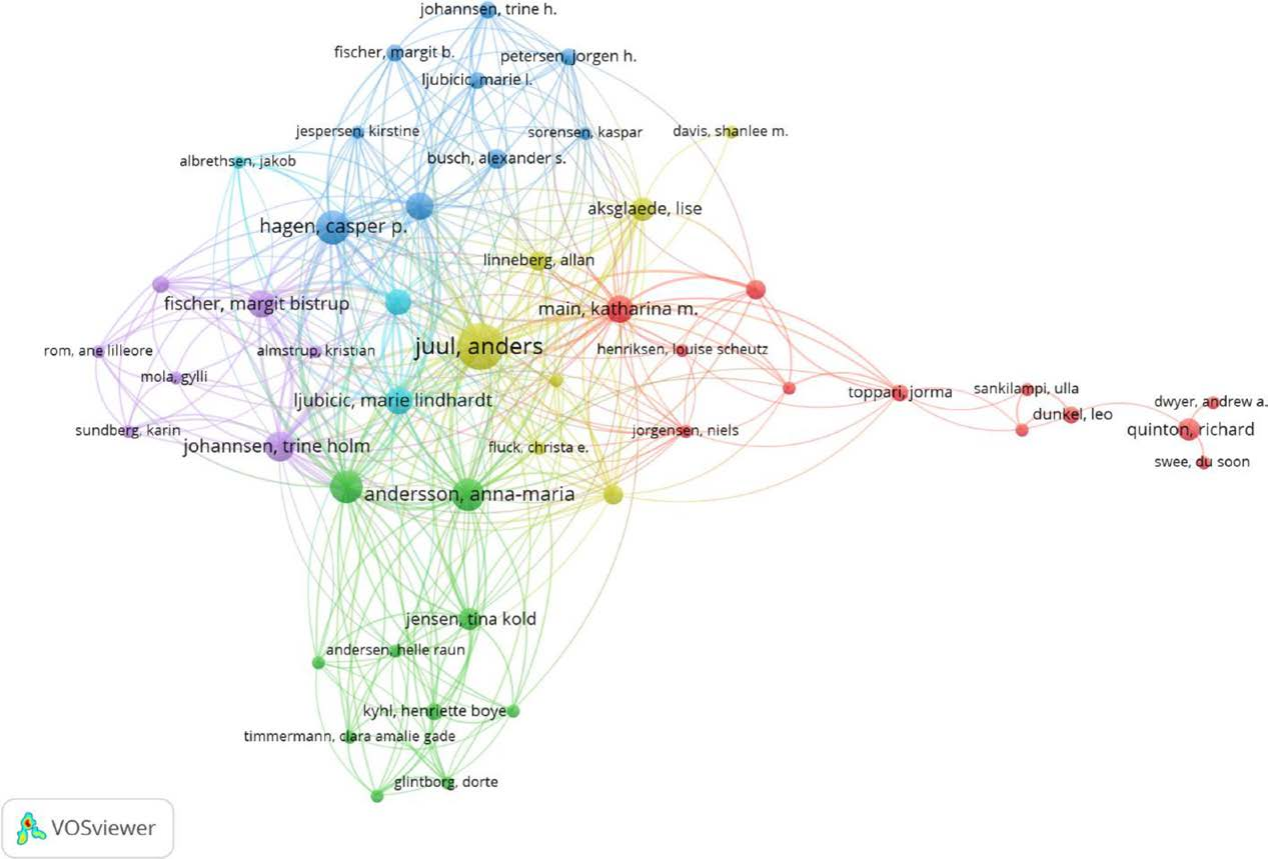
Co-authorship network among productive authors.

A total of 867 authors have contributed to publications in this field. As presented in Table 3, the top 10 most prolific authors are Juul A, Hadziselimovic F, Hagen CP, Verkauskas G, Andersson AM, Busch AS, Johannsen TH, Ljubicic ML, Fischer MB, and Frederiksen H, along with their annual publication trends. Notably, the majority of these leading authors are affiliated with the Department of Growth and Reproduction at Rigshospitalet in Denmark, with other significant contributions from institutions in Switzerland and Lithuania. By country of affiliation, 7 authors are based in Denmark, while one each are from Switzerland, Lithuania, Germany, and Finland (considering primary affiliation for authors with multiple institutions). Collaboration networks among these authors, visualized in Figure 5, indicate strong research partnerships primarily between teams in Denmark, Switzerland, and Germany.

**Table 3:** Top 10 most productive authors.

Authors	Articles	Articles fractionalized	H-index	G-index	M-index	TC	NP	PY start	Sources
Juul A	26	2.86	15	26	0.652	1048	26	2003	University of Copenhagen, Denmark.
Hadziselimovic F	22	6.73	13	20	0.591	426	22	2004	Cryptorchidism Research Institute, Switzerland.
Hagen CP	14	1.36	8	14	0.5	333	14	2010	Copenhagen University Hospital–Rigshospitalet, Denmark.
Verkauskas G	14	2.75	10	14	0.909	196	14	2015	Vilnius University Faculty of Medicine, Lithuania.
Andersson AM	12	1.11	9	12	0.563	477	12	2010	Copenhagen University Hospital–Rigshospitalet, Denmark.
Busch AS	12	1.17	7	12	0.7	179	12	2016	University of Münster, Germany.
Johannsen TH	12	1.13	7	12	0.875	233	12	2018	Copenhagen University Hospital–Rigshospitalet, Denmark.
Ljubicic ML	12	1.09	8	12	1	275	12	2018	Copenhagen University Hospital–Rigshospitalet, Denmark.
Fischer MB	11	1.05	5	10	1	111	11	2021	Copenhagen University Hospital–Rigshospitalet, Denmark.
Frederiksen H	11	1.04	8	11	1.333	163	11	2020	Copenhagen University Hospital–Rigshospitalet, Denmark.
Main KM	11	1.25	10	11	0.435	630	11	2003	Turku University Hospital, Finland.

PY = publication year, NP = number of publications, TC = total citations.

### 3.6. Analysis of the most active topics

#### 3.6.1. Keyword burst

**Figure 6.**
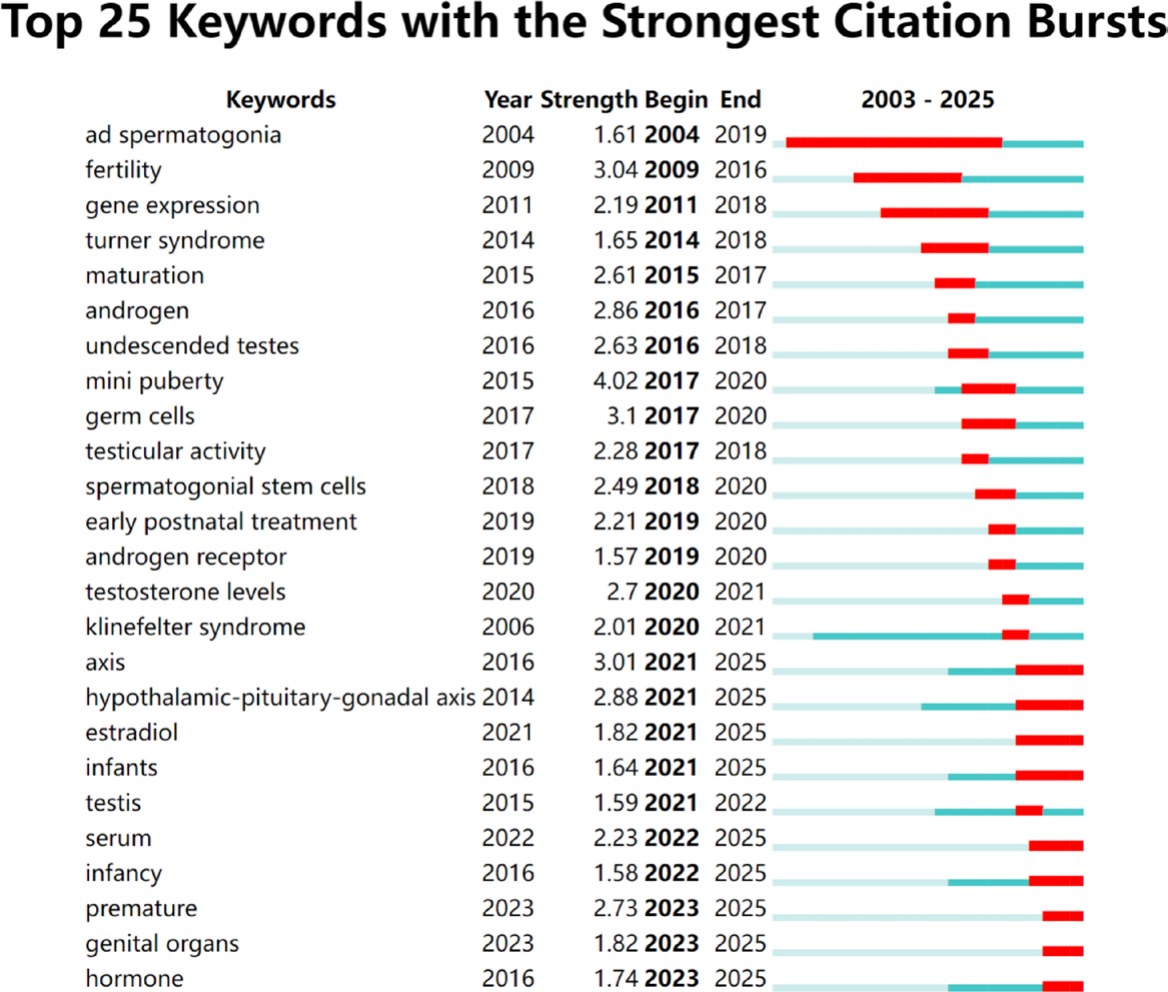
Top 25 keywords with the strongest citation bursts.

To elucidate research trends in minipuberty at a finer granularity, keyword burst analysis was conducted over the entire study period (2003–2025). Figure 6 displays the top 25 keywords with the strongest citation bursts. Among these, “ad spermatogonia” and “fertility” emerged as early focal points, with bursts beginning in 2004 and 2009 and burst strengths of 1.61 and 3.09, respectively. These keywords remained prominent until 2019, reflecting sustained research interest in the role of infantile ad spermatogonia activity and early hormonal profiles in fertility. The keyword “undescended testes” exhibited a burst strength of 2.63 between 2009 and 2013, indicating increased attention to the relationship between hormonal levels and cryptorchidism. Over time, the research focus evolved toward more specific mechanisms, such as the influence of growth hormone dynamics on male fertility. The term “minipuberty” itself showed a notable burst (strength: 4.02) during 2017 to 2020, highlighting growing interest in the dynamics of early hormonal changes and their correlation with pulsatile growth hormone secretion. In recent years, research emphasis has shifted to prevention and treatment strategies, encompassing themes such as “early postnatal treatment,” “androgen receptor,” “testosterone levels,” “axis,” “premature,” and “hormone.” Concurrently, diagnostic studies have focused on early hormonal assessment and the potential of hormone replacement therapy to mitigate fertility impairments linked to undescended testes.

#### 3.6.2. Keyword clustering analysis

**Figure 7.**
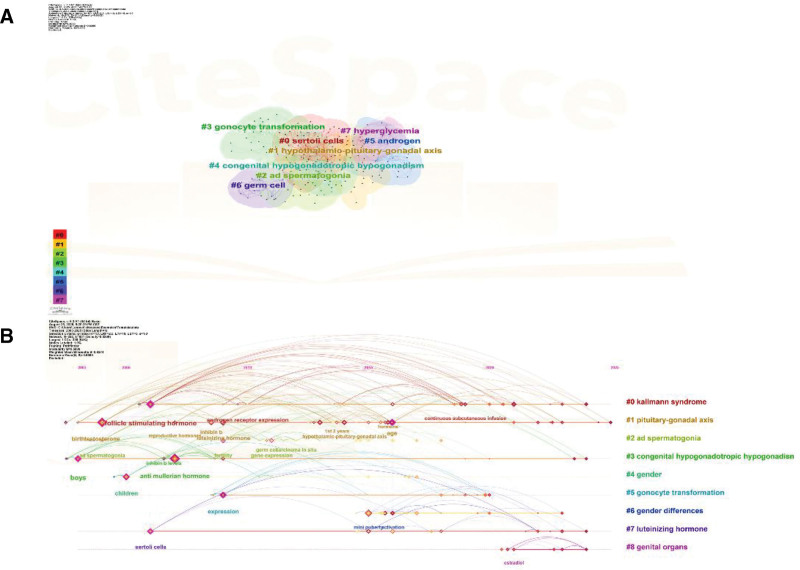
Keyword cluster snapshots. (A) Clustering form. (B) Timeline form.

Keyword analysis revealed strong intrinsic correlations among terms, with distinct thematic clusters emerging based on semantic affinity. The identification of these clusters provides an intuitive representation of subdomains within minipuberty research. A total of 8 clusters were identified (Fig. 7A), characterized by a modularity value (*Q*-value) of 0.4983 and a mean silhouette value (*S*-value) of 0.8066. A *Q*-value > 0.3 indicates a significant modular network structure, whereas an *S*-value > 0.7 suggests that the clusters are internally coherent and well-separated from each other, confirming the robustness of the thematic grouping. The primary thematic clusters include: #0 Sertoli cells, #1 HPG axis, #2 ad spermatogonia, #3 gonocyte transformation, #4 congenital hypogonadotropic hypogonadism, #5 androgen, #6 germ cell, and #7 hyperglycemia. These clusters encompass a broad spectrum of topics, underscoring the multidisciplinary scope of current research.

Furthermore, a timeline visualization derived from keyword co-occurrence clusters offers a comprehensive perspective on the evolution of research themes (Fig. 7B). This representation charts the chronological progression of major topics, with node size corresponding to keyword frequency, thereby emphasizing trends and shifts in research emphasis over time. The timeline not only illustrates the breadth of minipuberty research but also elucidates its developmental trajectory and conceptual depth.

### 3.7. Analysis of the most cited

Citation analysis provides a quantitative measure of the academic impact of research articles, where citation frequency often reflects a publication’s recognition within the scholarly community. As summarized in Table 4, the 10 most-cited publications in this field are listed. Among these, the top 7 papers have each been cited over 100 times, with the 2 highest-ranked articles exceeding 200 citations.

**Table 4:** Top 10 most-cited publications.

Paper	DOI	Total citations
Kuiri-Hänninen T, 2014, Horm Res Paediat	10.1159/000362414	294
Aksglæde L, 2006, Hum Reprod Update	10.1093/Humupd/Dmi039	206
Aksglaede L, 2010, J Clin Endocr Metab	10.1210/Jc.2010-1207	196
Chellakooty M, 2003, J Clin Endocr Metab	10.1210/Jc.2002-021468	129
Lanciotti L, 2018, Front Endocrinol	10.3389/Fendo.2018.00410	127
Hines M, 2015, Biol Sex Differ	10.1186/S13293-015-0022-1	127
Teerds KJ, 2015, Hum Reprod Update	10.1093/Humupd/Dmv008	122
Johannsen TH, 2018, J Clin Endocr Metab	10.1210/Jc.2018-00482	97
Li G, 2017, Dev Psychol	10.1037/Dev0000281	85
Dunkel L, 2014, Eur J Endocrinol	10.1530/EJE-13-0894	85

## 4. Discussion

This bibliometric analysis provides a systematic overview of the global research landscape on minipuberty from 2003 to 2025. Our findings reveal a field that has evolved from a niche area to a growing, collaborative, and clinically engaged domain of pediatric endocrinology.

The surge in annual publications since 2013 likely marks a pivotal period in which the clinical and developmental significance of minipuberty gained wider recognition, facilitated by advances in analytical techniques and the establishment of prospective birth cohorts. Geographically, the leadership of Denmark and the United States, coupled with the prominent role of the University of Copenhagen, highlights the concentration of expertise and sustained investigative efforts in Northern Europe. The strong international collaboration network, particularly within Europe, suggests a shared recognition of research priorities and a synergistic approach to addressing them. The United Kingdom’s notably high average citation impact, despite a moderate publication volume, underscores the influence of its contributions, which may include seminal reviews or pivotal studies that have shaped the field’s conceptual framework.

The thematic evolution traced by our keyword analysis reflects the maturation of the field. Early research (2004–2013) established the foundational link between the postnatal hormonal surge, gonocyte transformation into ad spermatogonia, and future male fertility,^[10,11]^ while concurrently exploring its disruption in conditions such as cryptorchidism and congenital hypogonadotropic hypogonadism.^[10,12]^ The subsequent shift shows a clear transition toward translational science. Research focus has expanded to include the mechanisms of endocrine disruption by environmental chemicals,^[13–15]^ the exploration of novel molecular pathways such as the “backdoor” androgen biosynthesis route, and the critical evaluation of therapeutic interventions like gonadotropin-releasing hormone analogs.

Seminal work has significantly advanced the understanding of minipuberty. Subsequent research on disorders such as Klinefelter syndrome revealed impaired minipubertal hormone levels.^[16]^ Further studies linked minipubertal hormonal activity to the development of secondary sexual characteristics.^[17]^ Clinical correlations include strong associations between disrupted minipuberty and cryptorchidism or micropenis.^[18]^ Exogenous factors – such as topical antifungals,^[19]^ phthalates, and phenolic compounds^[13–15]^ – have also been shown to interfere with hormonal regulation during this period.

Current research is advancing from descriptive hormonal profiling toward elucidating the molecular mechanisms that regulate the HPG axis.^[20]^ This shift is reflected in our keyword clustering (e.g., cluster #1 related to the HPG axis) and recent keyword bursts (e.g., “kisspeptin,” “neurokinin B”). Furthermore, the “backdoor pathway” as the primary route for androgen biosynthesis during minipuberty in male infants corresponds thematically to a distinct keyword cluster (#5 androgen).^[21]^ Keyword analysis also indicates that, beyond intrinsic regulatory mechanisms, external and maternal factors – such as vitamin D status,^[22]^ maternal gestational metabolic^[23]^ and endocrine conditions – as well as the programming effects of minipuberty on long-term metabolic health and neurodevelopment,^[24]^ are emerging research directions.

Future research should prioritize large-scale longitudinal cohorts tracking individuals from minipuberty through adulthood to clarify links between early hormonal patterns and long-term health outcomes. High-quality randomized controlled trials and meta-analyses are needed to evaluate interventions for related disorders. Minimally invasive sampling methods and sensitive assays will facilitate clinical translation, while computational endocrinology – using machine learning to decipher complex hormonal data – could enable personalized prediction and intervention. Ethical considerations and family-centered outcomes must remain central as technologies advance.

Several limitations warrant acknowledgment. First, reliance on English-language articles from WoSCC may omit relevant studies in other languages or databases. Second, citation-based metrics reflect scholarly influence but not necessarily clinical importance or quality. Finally, rapidly evolving trends may be underrepresented due to the limited citation accrual of recent publications. Our analysis was conducted using the standard functions and settings of established bibliometric software (Bibliometrix, VOSviewer, CiteSpace). While this ensures alignment with common analytical practices in the field, it inherently limits full computational reproducibility outside these software platforms.

## 5. Conclusion

This study delineates the landscape of minipuberty research, highlighting its growth from a niche interest to an active, collaborative, and clinically engaged field. Future research should prioritize longitudinal studies linking minipuberty biomarkers to long-term reproductive outcomes, refine diagnostic and therapeutic protocols for associated endocrine disorders, and expand multidisciplinary collaborations to integrate genetic, metabolic, and environmental perspectives. Such efforts will be essential to fully elucidate the physiological and pathological significance of this early developmental period.

## Author contributions

**Conceptualization:** Yi Jiang, Wen Zhong, Xiaojing Zhong.

**Data curation:** Kaiqin Jin, Wen Zhong.

**Methodology:** Kaiqin Jin.

**Software:** Kaiqin Jin.

**Visualization:** Kaiqin Jin.

**Writing – original draft:** Yi Jiang, Xiaojing Zhong.

**Writing – review & editing:** Yi Jiang, Wen Zhong, Xiaozhu Shen, Xiaojing Zhong.

## References

[1] Bangalore Krishna K Witchel SF. Normal puberty. Endocrinol Metab Clin North Am. 2024;53:183–94. 38677861 10.1016/j.ecl.2024.01.001

[2] Kaiserman KB Nakamoto JM Geffner ME McCabe ER. Minipuberty of infancy and adolescent pubertal function in adrenal hypoplasia congenita. J Pediatr. 1998;133:300–2. 9709728 10.1016/s0022-3476(98)70242-2

[3] Massa G de Zegher F Vanderschueren-Lodeweyckx M. Serum levels of immunoreactive inhibin, FSH, and LH in human infants at preterm and term birth. Biol Neonate. 1992;61:150–5. 1610942 10.1159/000243737

[4] Johannsen TH Main KM Ljubicic ML. Sex differences in reproductive hormones during mini-puberty in infants with normal and disordered sex development. J Clin Endocrinol Metab. 2018;103:3028–37. 29917083 10.1210/jc.2018-00482

[5] Kaplan SL Grumbach MM. The ontogenesis of human foetal hormones. II. Luteinizing hormone (LH) and follicle stimulating hormone (FSH). Acta Endocrinol (Copenh). 1976;81:808–29. 946570 10.1530/acta.0.0810808

[6] Lucaccioni L Trevisani V Boncompagni A Marrozzini L Berardi A Iughetti L. Minipuberty: looking back to understand moving forward. Front Pediatr. 2020;8:612235. 33537266 10.3389/fped.2020.612235 PMC7848193

[7] Becker M Hesse V. Minipuberty: why does it happen? Horm Res Paediatr. 2020;93:76–84. 32599600 10.1159/000508329

[8] Chellakooty M Schmidt IM Haavisto AM. Inhibin A, inhibin B, follicle-stimulating hormone, luteinizing hormone, estradiol, and sex hormone-binding globulin levels in 473 healthy infant girls. J Clin Endocrinol Metab. 2003;88:3515–20. 12915629 10.1210/jc.2002-021468

[9] Aria M Cuccurullo C. bibliometrix: an R-tool for comprehensive science mapping analysis. J Inform. 2017;11:959–75.

[10] Hadziselimovic F Emmons LR Buser MW. A diminished postnatal surge of Ad spermatogonia in cryptorchid infants is additional evidence for hypogonadotropic hypogonadism. Swiss Med Wkly. 2004;134:381–4. 15340882 10.4414/smw.2004.10575

[11] Hadziselimovic F Zivkovic D Bica DTG Emmons LR. The importance of mini-puberty for fertility in cryptorchidism. J Urol. 2005;174:1536–9; discussion 1538. 16148647 10.1097/01.ju.0000181506.97839.b0

[12] Koskenniemi JJ Virtanen HE Wohlfahrt-Veje C. Postnatal changes in testicular position are associated with IGF-I and function of Sertoli and Leydig cells. J Clin Endocrinol Metab. 2018;103:1429–37. 29408984 10.1210/jc.2017-01889

[13] Mueller ML Busch AS Ljubicic ML. Urinary concentration of phthalates and bisphenol A during minipuberty is associated with reproductive hormone concentrations in infant boys. Int J Hyg Environ Health. 2023;250:114166. 37058994 10.1016/j.ijheh.2023.114166

[14] Muerkoster AP Frederiksen H Juul A. Maternal phthalate exposure associated with decreased testosterone/LH ratio in male offspring during mini-puberty. Odense Child Cohort. Environ Int. 2020;144:106025. 32798799 10.1016/j.envint.2020.106025

[15] Jensen TK Andersson AM Main KM. Prenatal paraben exposure and anogenital distance and reproductive hormones during mini-puberty: a study from the Odense Child Cohort. Sci Total Environ. 2021;769:145119. 33477047 10.1016/j.scitotenv.2021.145119

[16] Aksglæde L Wikström AM Rajpert-De Meyts E Dunkel L Skakkebæk NE Juul A. Natural history of seminiferous tubule degeneration in Klinefelter syndrome. Hum Reprod Update. 2006;12:39–48. 16172111 10.1093/humupd/dmi039

[17] Henriksen LS Hagen CP Assens M. Genetic variations in FSH action affect sex hormone levels and breast tissue size in infant girls: a pilot study. J Clin Endocrinol Metab. 2016;101:3191–8. 27270476 10.1210/jc.2016-1672

[18] Khadilkar V Mondkar SA. Micropenis. Indian J Pediatr. 2023;90:598–604. 37079255 10.1007/s12098-023-04540-w

[19] Andreasen SM Iversen AP Lund LC. Maternal application of tropical antifungal medication is associated with reduced steroid hormone levels during minipuberty and shorter anogenital distance in offspring from 3 months to 9 years of age: Odense Child Cohort. Reprod Toxicol. 2025;137:109007. 40706775 10.1016/j.reprotox.2025.109007

[20] Hadziselimovic F Gegenschatz-Schmid K Verkauskas G. Gene expression changes underlying idiopathic central hypogonadism in cryptorchidism with defective mini-puberty. Sex Dev. 2016;10:136–46. 27561106 10.1159/000447762

[21] Dhayat NA Dick B Frey BM d’Uscio CH Vogt B Flück CE. Androgen biosynthesis during minipuberty favors the backdoor pathway over the classic pathway: insights into enzyme activities and steroid fluxes in healthy infants during the first year of life from the urinary steroid metabolome. J Steroid Biochem Mol Biol. 2017;165:312–22. 27471148 10.1016/j.jsbmb.2016.07.009

[22] Kilinc S Atay E Ceran O Atay Z. Evaluation of vitamin D status and its correlation with gonadal function in children at mini-puberty. Clin Endocrinol (Oxf). 2019;90:122–8. 30229999 10.1111/cen.13856

[23] Kowalcze K Krysiak R Obuchowicz A. The impact of maternal hypothyroidism during pregnancy on minipuberty in boys. J Clin Med. 2023;12:7649. 38137718 10.3390/jcm12247649 PMC10744195

[24] Kowalcze K Burgio S Ott J Gullo G Zaami S Krysiak R. The impact of maternal gestational diabetes mellitus on minipuberty in boys. Nutrients. 2024;16:4145. 39683537 10.3390/nu16234145 PMC11644001

